# New Hospitals

**Published:** 1916-03

**Authors:** 


### New Hospitals.

A sanitarium has recently been established in Columbus known as the Columbus Santarium. It will be a thoroughly equipped institution, fully prepared to care for medical, surgical and obstetrical cases.

Dr. F. S. Groner, pastor of the Columbus Street Baptist Church, has accepted the position as chairman of the $100,000 Baptist Sanitarium for Waco. Efforts will be made to raise $100,000 immediately with which the first building will be installed.

The rock building west of the Central Hotel, Belton, will be turned into an up-to-date sanitarium. All the physicians of the city have pledged their support.

Contract has been let for the annexation to the present hospital building, corner of Walnut and Fifth Streets, Dallas. It is stated that the additions to be made will call for an expenditure of $15,000.

Plans are being drawn for a new $150,000 hospital for El Paso.

Plans are now under way to transform the United States Mint Building, New Orleans, La., into a hospital for the treatment of persons suffering from tuberculosis.

Mrs. John W. Gates has recently given $5000 for additional equipment for the Mary Gates Hospital, Beaumont.

Corpus Christi is to have a new sanitarium, completely and modernly equipped, to be located on Central wharf, several hundred yards out over the bay.

Arrangements have been completed for the beginning of a campaign to raise $500,000 for the establishment of a hospital to be operated in connection with the medical department of Texas Christian University, Fort Worth, and which will have an endowment fund of $1,000,000.

The question of a hospital for Canadian has again been raised, and under the new conditions we have every reason to believe that under the new conditions the proposition is well launched and that the near future will see positive evidences of realization.

